# Effect of frequent interruptions of prolonged sitting on self-perceived levels of energy, mood, food cravings and cognitive function

**DOI:** 10.1186/s12966-016-0437-z

**Published:** 2016-11-03

**Authors:** Audrey Bergouignan, Kristina T. Legget, Nathan De Jong, Elizabeth Kealey, Janet Nikolovski, Jack L. Groppel, Chris Jordan, Raphaela O’Day, James O. Hill, Daniel H. Bessesen

**Affiliations:** 1 Anschutz Health and Wellness Center, University of Colorado Anschutz Medical Campus, Aurora, CO USA; 2 Division of Endocrinology, Metabolism and Diabetes, Department of Medicine, Anschutz Medical Campus, University of Colorado School of Medicine, 12801 East 17th Avenue Mail Stop: 8106, Aurora, CO 80045 USA; 3 Universite de Strasbourg, IPHC, Strasbourg, France; 4 CNRS; UMR7178, Strasbourg, France; 5 Department of Psychiatry, Anschutz Medical Campus, University of Colorado School of Medicine, Aurora, CO USA; 6 Johnson & Johnson Consumer Inc., Skillman, NJ USA; 7 Johnson & Johnson Human Performance Institute, Orlando, FL USA; 8 Johnson & Johnson Health and Wellness Solutions, New Brunswick, NJ USA

**Keywords:** Sedentary behavior, Sitting, Physical activity, Exercise, Fatigue, Appetite, Catecholamines

## Abstract

**Background:**

While physical activity has been shown to improve cognitive performance and well-being, office workers are essentially sedentary. We compared the effects of physical activity performed as (i) one bout in the morning or (ii) as microbouts spread out across the day to (iii) a day spent sitting, on mood and energy levels and cognitive function.

**Methods:**

In a randomized crossover trial, 30 sedentary adults completed each of three conditions: 6 h of uninterrupted sitting (SIT), SIT plus 30 min of moderate-intensity treadmill walking in the morning (ONE), and SIT plus six hourly 5-min microbouts of moderate-intensity treadmill walking (MICRO). Self-perceived energy, mood, and appetite were assessed with visual analog scales. Vigor and fatigue were assessed with the Profile of Mood State questionnaire. Cognitive function was measured using a flanker task and the Comprehensive Trail Making Test. Intervention effects were tested using linear mixed models.

**Results:**

Both ONE and MICRO increased self-perceived energy and vigor compared to SIT (*p* < 0.05 for all). MICRO, but not ONE, improved mood, decreased levels of fatigue and reduced food cravings at the end of the day compared to SIT (*p* < 0.05 for all). Cognitive function was not significantly affected by condition.

**Conclusions:**

In addition to the beneficial impact of physical activity on levels of energy and vigor, spreading out physical activity throughout the day improved mood, decreased feelings of fatigue and affected appetite. Introducing short bouts of activity during the workday of sedentary office workers is a promising approach to improve overall well-being at work without negatively impacting cognitive performance.

**Trial registration:**

NCT02717377, registered 22 March 2016.

## Background

The industrial and technological revolutions have profoundly altered the occupational conditions of modern societies. While the majority (60–70 %) of workers in the Organization for Economic Co-operation and Development (OECD) countries had blue-collar jobs in the 1970s, by the 1990s about 60–70 % were employed in jobs characterized by work in office environments [1]. These developments have had an overall beneficial impact on occupational health. However, new job demands, new working methods, and the increased need for processing and analyzing information may have placed a high demand on workers and may have increased mental stress and detrimentally impacted well-being and mood [2].

Physical activity is known to positively affect cognitive performance, concentration, well-being and mood [3–7]. However, the expansion of service occupations has reduced physical activity by 20 % at the workplace since 1960, which could be 35 % by 2030 [8]. For those working in offices, 65–75 % of their work time is spent sitting, with time spent sitting at work accounting for more than half of the total daily sitting time on work days [9–11]. Only recently has exercise been proposed as a worksite strategy to improve performance, concentration and satisfaction at work [12].

While it is well-established that 30 min of moderate-intensity physical activity per day for at least 5 days a week can have a beneficial impact on health [13], the dose needed to improve well-being is less clear. Nevertheless, it is impractical for most people to identify the time to participate in a 30-min bout of exercise during the workday. Because of competing interests, most physically active adults exercise before or after their workday. This strategy may not, however, have the same beneficial effects on energy levels, mood and cognitive function as physical activity performed throughout the workday. Breaking down 30 min of exercise into short bouts of exercise that can be performed during 5-min breaks may be a more feasible approach that may have a more lasting impact over the workday on energy levels, mood, and cognitive performance.

To test this idea, we conducted a randomized cross-over study comparing the effects of 5-min bouts of moderate-intensity physical activity performed every hour for 6 h to a 30-min continuous bout of moderate-intensity physical activity performed early in the morning, on self-reported energy, cognitive function, fatigue and mood levels in healthy non-obese sedentary adults. These conditions were also compared to a sedentary control condition. We also measured the effects of these conditions on urinary concentration of epinephrine, norepinephrine and cortisol, which are indicators of physiological stress, as well as on urinary levels of dopamine, a neurotransmitter involved in the regulation of cognition and attention [14]. Because perceived hunger and appetite have been reported to modify cognitive function and feelings of fatigue and mood [15–17], we further examined changes in perceived hunger and appetite throughout the day in each condition.

## Methods

### Participants

A total of 30 participants were recruited from a population of healthy, sedentary (self-reporting sitting time > 9 h/day), non-obese (body mass index, BMI between 18.5 and 29.9 kg/m^2^) adult men and women (M = 9, *F* = 21) who were between 25 and 50 years of age and who did not report meeting levels of physical activity recommended by current guidelines (self-reported moderate-to-vigorous physical activity < 150 min/week). Subjects were recruited from newspaper advertisements, public service announcements, and flyers in the Denver and Aurora areas. Subjects were excluded if they reported drinking more than three caffeinated beverages per day, smoked, had a history of cardiovascular disease, uncontrolled hypertension, or if they used medications affecting weight, energy intake or energy expenditure. Females were excluded if they planned to get pregnant, or were currently pregnant, lactating, less than 6 months post-partum or post-menopausal. Alcohol intake was not an exclusion criteria.

### Study design

Following a screening visit, each subject completed three separate 1-day trial conditions, administered in random order: (i) uninterrupted sitting (SIT), (ii) uninterrupted sitting plus one bout of 30 min of moderate-intensity physical activity in the morning (ONE); (iii) uninterrupted sitting plus six 5-min microbouts of moderate-intensity physical activity performed every hour for 6 h (MICRO). The two physically active conditions (ONE and MICRO) were designed to last 30 min total each and to expend an equal amount of energy. Study visits were conducted at the Anschutz Health and Wellness Center (AHWC) on the Anschutz Medical Campus of the University of Colorado. Every participant completed written informed consent following a detailed explanation of study procedures. This study was approved by the Western Institutional Review Board.

### Screening visit

Once participants passed the initial phone screening, they were invited to the AHWC for an in-person screening visit that consisted of physical measures including height, weight and blood pressure, to assure study qualification. The short version of the International Physical Activity Questionnaire (IPAQ) [18] was completed at screening to assess study eligibility based on inclusion criteria for habitual physical activity (<150 min per week moderate-to-vigorous physical activity) and time spent sedentary (>9 h spent sedentary per day). Subjects also completed questionnaires to assess socio-economic status and mood (Beck Depression Inventory-II [BDI-II]) [19]. Subjects then performed an incremental-speed test on a motorized treadmill, with increasing increments of 0.3 mph and 0.5 % incline every 2 min. For each level, subjects rated their perceived effort on a Borg scale from 6 (“very light”) to 20 (“maximal exertion”). The aim was to identify the speed that each participant associated with a level of effort between 12 and 13 (“somewhat hard”). This was the treadmill speed that was used for the activity study days. Subjects were then given a physical activity monitor (ActivPAL; PAL Technologies Ltd, Glasgow, Scotland) to measure daily time spent sitting/lying, standing, and walking, in addition to sit-to-stand and stand-to-sit transition counts and steps counts, for 1 week to objectively determine habitual physical activity levels. Participants were instructed to wear the monitor on their right leg at all times except when sleeping or participating in water-based activities.

### Study protocol

Subjects completed the three study days on a Tuesday, Wednesday, or Thursday, to minimize any effects from weekend activity levels. Study visits were separated by a minimum of 1-week wash out period. The three study conditions were as follows:

- ***SIT*** **.** *Uninterrupted sitting*: Subjects remained seated all day except to rise from the chair to void.
- ***ONE*** **.** *Sitting + one bout of activity*: Subjects remained seated all day, except to rise from the chair to void, and to perform one bout of 30-min moderate-intensity walking. Physical activity was performed at 0800, after measures of vitals and basal questionnaire assessments, but before breakfast.
- ***MICRO*** **.** *Sitting + microbursts of activity:* Subjects rose from the seated position every hour for 6-h from 0910 to 1430 to complete 5-min bouts of moderate-intensity walking, yielding a total activity time of 30-min.

When sitting, participants were allowed to read, use a computer and watch TV. For the conditions ONE and MICRO, walking bouts took place on a motorized treadmill.

### Diet

Subject’s diets were not controlled the night before study days. However, to control the effects of diet, subjects were fed a standardized breakfast and lunch on each study day. The energy requirements for the three study days were calculated based on an estimate of resting metabolic rate (RMR) derived from the Mifflin-St Jeor equation. RMR was then multiplied by a conservative activity factor of 1.3, representative of a sedentary lifestyle. Energy intake during the two physically active conditions was the same as that during the sedentary control condition, resulting in a slight energy deficit by design. All meals were prepared by the AHWC metabolic kitchen and had the same macronutrient composition (15 % protein, 55 % carbohydrate 30 % fat). Breakfast meals provided 25 % and lunch meals provided 30 % of the total estimated caloric needs. Subjects were required to consume all food provided and no additional food, other than non-caloric beverages, was permitted. Subjects who habitually consumed coffee or tea were allowed to have a maximum of two 8-ounce servings at breakfast; all other beverages were non-caffeinated. The amount of caffeine consumption was matched for each subject across each of the three conditions.

### Study day

**Fig. 1:**
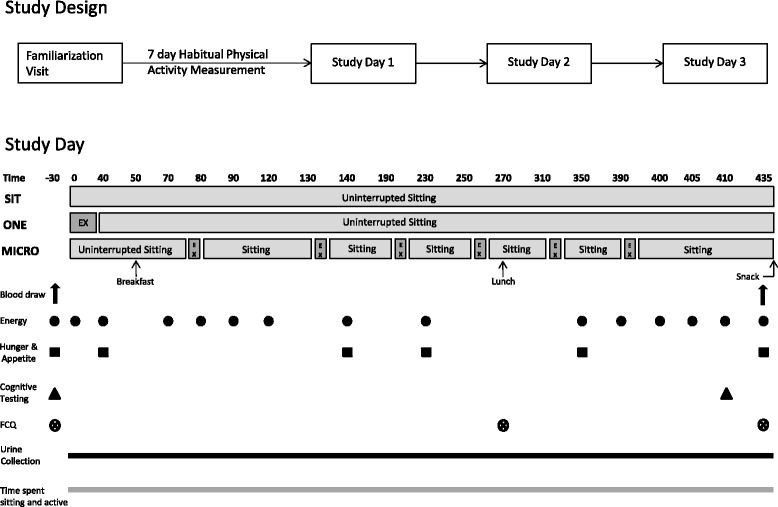
Study Protocol. CTMT: Comprehensive Trail Making Test; FCQ: Food craving questionnaires; MICRO. Sitting + microbursts of activity; ONE. Sitting + one bout of activity; POMS: Profile of Mood States

The protocol is summarized in Fig. 1. For each study day (~10 h), subjects arrived via passive transportation (e.g., car) at the AHWC at 0700 in a 10-h fasted state and were provided access to the closest parking from the AHWC (less than 50 m walking distance). After collecting baseline vital signs, subjects were asked to void. ActivPAL and Actiheart (Camntech CamNtech Ltd and CamNtech Inc., UK) devices were placed on the right leg and chest of the participants, respectively, to objectively determine physical activity levels. Self-perceived energy and mood were measured by using visual analogue scales (VAS) as described below at baseline, 0800, 0840, 0850, 0910, 0920, 0930, 1000, 1020, 1150, 1350, 1430, 1440, 1445, 1450 and 1515. A modified version of the Profile of Mood States (POMS) was administered at baseline and 1450 to assess levels of vigor and fatigue (details below). Two cognitive tests (a flanker task and the Comprehensive Trail Making Test [CTMT]) were administered at the end of the day (1450), as detailed below. Perceived hunger and appetite were assessed at baseline, 0840, 1020, 1150, 1350 and 1515, by VAS. Self-perceived food craving sensation was measured by using the Food Cravings Questionnaire (FCQ) at 0840, 1230, and 1515. From 0800 to 1515, urine was collected throughout the day to measure creatinine, catecholamines, dopamine and cortisol (details below). At 1520, activity monitors were removed and subjects received a granola bar snack prior to leaving the AHWC.

### Perceived energy, mood and fatigue

A VAS was used to assess changes in self-perceived energy level and mood. Participants were told to consider the extremes of each rating as the most intense sensation they could imagine. Questions were presented one at a time on the screen of a tablet computer, accompanied by a 100 mm horizontal line. Participants read each question, then used a stylus to mark their response along the horizontal line. For Energy, the question was “*What is your energy level right now?*” with the left anchor being “Lowest Energy” and the right anchor “Highest Energy.” For Mood, the question was “*What is your mood level right now?*” with the left anchor being “Negative Mood” and the right anchor “Positive Mood.” Once a response to a question was recorded, the participant pressed “continue” to proceed to the next question. Participants could not revise previously recorded answers.

At baseline and at the end of each study day, a modified version of the POMS was used to further assess changes in feelings of vigor and fatigue [20]. The POMS consists of 65 Likert scale items that measure mood states. Only the POMS-Fatigue (POMS-F; *n* = 7 items) and the POMS-Vigor (POMS-V; *n* = 8 items) subscales were used in this study, to assess energy state. Scoring was on a 4-point Likert-type scale, from 0 = “Not at all” to 4 = “Extremely,” with summed scores calculated separately for the POMS-F and POMS-V.

### Cognitive performance

Participants completed two measures of cognitive performance on each study day. Inhibitory control was assessed using a modified Eriksen flanker task [21] in the afternoon of each study day. The task was presented on a computer, using E-Prime 2.0 software (Psychology Software Tools, Inc., Sharpsburg, PA). In each trial, a series of five white arrows were presented in the center of a black background. Some trials were “congruent” trials, in which the middle arrow (the “target”) was pointed in the same direction (left or right) as the other four arrows (e.g., > > > > >). In “incongruent trials”, the target arrow was pointed in the opposite direction from the other four arrows (e.g., > > < > >). Participants were asked to identify, via key press, whether the target arrow was pointing to the left or to the right, as quickly and accurately as possible. Response times and accuracy for congruent and incongruent trials were recorded. Interference scores were also calculated for response time and accuracy (incongruent score – congruent score), which reflect performance differences between congruent and incongruent trials.

Participants also completed the CTMT in the afternoon of each study day. The CTMT assesses attention and cognitive flexibility through five visual search and sequencing tasks [22]. In each of the five subtests, participants are asked to draw a continuous line to connect letters, numbers and words in a specified order. The score for each subtest is the time to completion. A CTMT composite index score was calculated by summing the raw time scores for each of the five subtests, then converting the total time score into a standardized T-score according to the participant’s age.

### Appetite ratings

Appetite was assessed by using VAS measures and the FCQ [23]. Appetite VAS measures were similar to those described for the energy and mood measures. Participants were presented with the following questions: “*How hungry do you feel?”,* “*How full do you feel?*”, and “*How much food do you think you could eat right now?*” Questions were accompanied by 100 mm horizontal lines, which were anchored at the left by “Not at all” or “Nothing at all,” and at the right by “Extremely” or “A large amount.”

The FCQ was administered prior to breakfast, lunch and snack to measure hunger level and how much food the participant was craving at that moment. The survey consists of 15 questions, with responses indicated on a 5-point Likert scale, anchored by 1 = “Strongly Disagree” and 5 = “Strongly Agree”. An example question is *“I know I’m going to keep on thinking about one of my favorite foods until I actually have it.”*

### Urinary catecholamines, cortisol and dopamine

Urinary catecholamines, cortisol and dopamine were measured by the core laboratory of the University of Colorado Hospital, by liquid chromatography-tandem mass spectrometry. They were corrected for creatinine excretion as measured by the Jaffe method also run by the University of Colorado Hospital.

### Statistical analysis

Data are expressed as mean ± SD, unless otherwise stated. Statistical analyses were performed with SPSS software (version 22.0, IBM Corp, Armonk, NY). Time course of perceived energy, mood, hunger and appetite were analyzed using linear mixed models with condition, time and condition-by-time as fixed effects, time as a repeated measure, and subjects as a random effect. A post-hoc Bonferroni test was then used to examine the differences at each time point within each condition. Self-reported energy, hunger, appetite and mood data points were used to calculate areas under the curve (AUC) over the time period of measurement. AUCs and urine hormones were analyzed using linear mixed models with condition as a fixed effect and repeated measure, and subjects as a random effect, followed by post-hoc Bonferroni test to account for multiple comparisons. Statistical adjustments for sequence and period were made. Pearson correlation coefficients were calculated to examine the relationships between the primary outcomes, i.e. self-perceived energy, mood and fatigue levels, appetite ratings and urinary hormones concentration. An alpha level of 0.05 was used for all statistical tests.

## Results

### Participant characteristics

The characteristics of the participants are displayed in Table 1. Nine males and 21 females with an average age of 31 ± 6 years and BMI of 23.8 ± 3.4 kg/m^2^ completed the study. On the physically active condition days (ONE and MICRO), subjects walked on a treadmill at an average pace of 3.6 ± 0.3 mph and a 5.4 ± 1.1 % grade.

**Table 1:** Subjects’ characteristics

N (Male/Female)	9/21
Age (yr)	30 ± 5.6
Height (m)	1.7 ± 0.1
Weight (kg)	63.4 ± 9.8
BMI (kg/m^2^)	23.0 ± 2.4
IPAQ-derived vigorous activity (minutes/week)	33 ± 100
IPAQ-derived moderate activity (minutes/week)	252 ± 356
IPAQ-derived Sitting (minutes/week)	1045 ± 266
Beck II Score (score range 0–63)	3.8 ± 4.1

Mean +/- SD

### Activity and heart rate

Time spent sitting, standing and stepping, as well as the daily heart rate measured during each of the three conditions, are reported in Table 2. The percent time spent sitting during the study day decreased from 93 ± 6 % in the SIT condition to 84 ± 10 % (mean difference = 9.6 ± 1.7, 95 % CI [5.5; 13.8], *p* < 0.0001) and 85 ± 4 % (mean difference = 8.2 ± 1.7, 95 % CI [4.0; 12.4], *p* < 0.0001) in the ONE and MICRO conditions, respectively. In contrast, the time spent stepping and the number of steps significantly increased in both ONE and MICRO conditions (*p* < 0.0001 for all). Both the number of steps and the time spent stepping were greater in MICRO compared to ONE (*p* < 0.001 for both). Furthermore, the physical activity conditions significantly raised the mean heart rate over the day from average 70.2 ± 9.7 bpm in SIT to 78.3 ± 9.9 and 80.3 ± 11.6 bpm in ONE and MICRO, respectively (*p* < 0.0001 for both).

**Table 2:** Activity and daily heart rate

	SIT	ONE	MICRO
Sitting (h)	7.74 ± 0.56	6.99 ± 0.93^b^	7.14 ± 0.46^b^
Standing (h)	0.45 ± 0.46	0.64 ± .81	0.45 ± 0.32
Stepping (h)	0.10 ± 0.04	0.71 ± 0.07^b^	0.78 ± 0.09^a, c^
Sitting (%)	93.42 ± 5.68	83.79 ± 10.21^b^	85.30 ± 4.15^a^
Standing (%)	5.40 ± 5.65	7.72 ± 10.01	5.36 ± 3.82
Stepping (%)	1.19 ± 0.48	8.47 ± 0.72^b^	9.34 ± 1.07^a, c^
Step count	418 ± 190	4715 ± 540^b^	5086 ± 610^a, c^
Daily Heart Rate (bpm)	70.2 ± 9.7	78.3 ± 9.9^b^	80.3 ± 11.6^a^

Mean +/- SD, ^a^ *P* < 0.05 SIT versus MICRO, ^b^ *P* < 0.05 SIT versus ONE, ^c^ *P* < 0.05 ONE versus MICRO

*SIT* uninterrupted sitting condition, *ONE* uninterrupted sitting plus one continuous 30-min bout of moderate intensity treadmill walking, *MICRO* uninterrupted sitting plus six 5-min bouts of moderate intensity treadmill walking, performed every hour for 6 h

### Perceived energy and mood levels

**Fig. 2:**
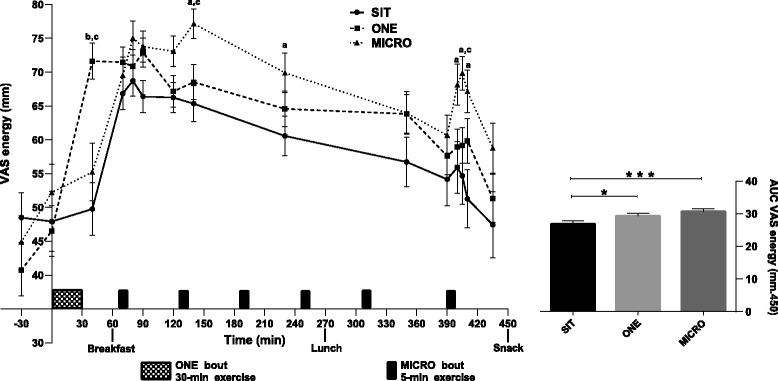
Self-perceived energy level over the day (*Left*) and area under the curve (AUC; *Right*) in uninterrupted sitting (SIT), uninterrupted sitting plus one continuous 30-min bout of moderate intensity treadmill walking (ONE), and uninterrupted sitting plus six 5-min bouts of moderate intensity treadmill walking, performed every hour for 6 h (MICRO), in healthy adults (*n* = 30). Changes over the day and between conditions, as well as differences in AUC, were tested by using a linear mixed model: Condition effect: *p* < 0.0001, Time effect: *p* < 0.0001 and Condition-by-time effect: *p* < 0.0001. Bonferroni post-hoc results: ^a^ *P* < 0.05 SIT versus MICRO, ^b^ *P* < 0.05 SIT versus ONE, ^c^ *P* < 0.05 ONE versus MICRO. For the AUC graph: *p < 0.05; **p < 0.01

Perceived energy levels significantly changed across the day (main effect of time: *p* < 0.0001), as shown in Fig. 2. In the SIT condition, perceived energy level peaked immediately after breakfast and then declined through the day back to the baseline value. Both physical activity conditions altered this time course (Treatment-by-time: *p* < 0.0001). In the ONE condition, immediately after the single bout of exercise (0840, as per Fig. 1), participants reported higher energy levels than those reported in both SIT and MICRO conditions at the same time point (*p* < 0.05 for both). After this, there were no statistically significant differences in energy levels between the SIT and ONE conditions, suggesting that the effect of the one bout of activity did not last over the day. In the MICRO condition, the first 5-min bout of physical activity had no significant effect. After the second bout, however, perceived energy level was greater compared to both SIT and ONE conditions (*p* < 0.05 for both). When measuring energy level immediately after the last 5-min bout of exercise, participants reported a higher energy level in the MICRO as compared to the feeling of energy reported in the SIT condition (1440, 1445; 1450) and even higher than that in the ONE condition (1445, *p* < 0.05 for all). Energy level AUCs were significantly increased by 15 ± 25 % and 16 ± 26 % in ONE (mean difference = −2583 ± 943, 95 % CI [−4909; −257], *p* = 0.03) and MICRO (mean difference = −3900 ± 987, 95 % CI [−6336; −1464], *p* = 0.001) conditions, respectively, compared to SIT. However, energy level AUCs were not significantly different between the two active conditions.

**Fig. 3:**
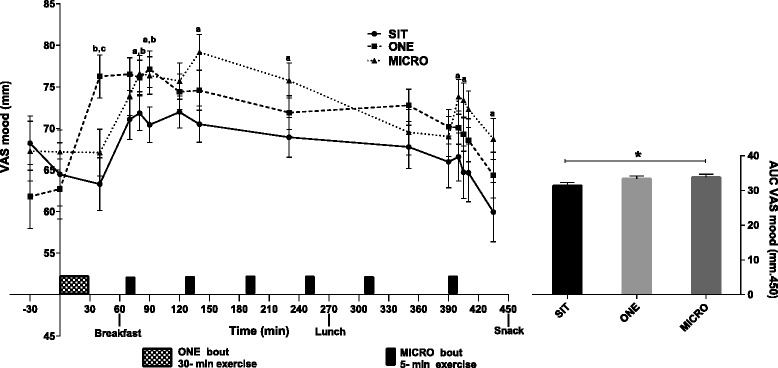
Self-perceived mood level over the day (*Left*) and area under the curve (AUC; *Right*) in uninterrupted sitting (SIT), uninterrupted sitting plus one continuous 30-min bout of moderate intensity treadmill walking (ONE), and uninterrupted sitting plus six 5-min bouts of moderate intensity treadmill walking, performed every hour for 6 h (MICRO), in healthy adults (*n* = 30). The changes over the day and between conditions, as well as differences in AUC, were tested by using a linear mixed model: Condition effect: *p* < 0.0001, Time effect: *p* < 0.0001 and Condition-by-time effect: *p* = 0.032. Bonferroni post-hoc results: ^a^ *P* < 0.05 SIT versus MICRO, ^b^ *P* < 0.05 SIT versus ONE, ^c^ *P* < 0.05 ONE versus MICRO. For the AUC graph: *p < 0.05

Changes in reported mood levels (VAS scale, with 0 = negative to 100 = positive) were overall similar to those reported for energy levels, as illustrated in Fig. 3. In SIT, mood levels increased after breakfast and gradually decreased to reach values lower than those reported at baseline by the end of the study day. Both physical activity interventions altered this profile (Treatment-by-time: *p* = 0.03). As with energy level, perceived mood level was significantly higher after the single bout of exercise in ONE compared to levels reported at this same time point in both SIT and MICRO conditions (*p* < 0.05 for both), but this beneficial effect lasted for only 1 h following exercise compared to the SIT condition (0920, 0930, *p* < 0.05 for both). Contrary to the results reported for energy, one bout of 5-min treadmill walking was sufficient to significantly improve mood compared to the level reported in SIT condition (*p* < 0.05), and as the bouts of activity continued through the day, this greater mood level was observed at almost every time point across the study day. As a result, mood AUC was significantly higher in the MICRO condition compared to SIT (mean difference = −2190 ± 965, 95 % CI [−4124; −257], *p* = 0.04). No significant differences were noted between the ONE and MICRO conditions.

### The fatigue-vigor scales

POMS-F and POMS-V scores were measured both at baseline and at the end of the study day (Table 3). As expected, no significant differences between the three conditions were noted for either POMS-F or POMS-V scores at baseline. At the end of the study day, participants reported feeling significantly more vigorous in both ONE (mean difference = −2.1 ± 0.7, 95 % CI [−3.9; −0.3], *p* = 0.01) and MICRO (mean difference = −2.8 ± 0.7, 95 % CI [−4.6; −1.1], *p* < 0.0001), compared to SIT. Specifically, participants felt more active, cheerful, alert, full of pep and vigorous in the MICRO condition as compared to SIT (*p* < 0.05 for all). They still felt more full of pep (*p* = 0.004) at the end of the day after one 30-min bout of physical activity in the morning. On the contrary, POMS-F score was significantly lower at the end of the MICRO day (mean difference = 2.0 ± 0.6, 95 % CI [0.5; 3.4], *p* = 0.004) compared to SIT. Specifically, subjects reported feeling less fatigued, weary, bushed and sluggish after walking 5 min every hour than when remaining seated the whole day (*p* < 0.05 for all). No statistical differences were noted for either the fatigue and vigor scales between the ONE and SIT conditions.

**Table 3:** POMS Fatigue and Vigor subscales and overall scores, food craving questionnaire and urinary hormone concentrations

Study outcomes		SIT	ONE	MICRO
Fatigue	Overall Morning	4.0 ± 3.7	4.4 ± 4.0	3.7 ± 4.2
	Overall Afternoon	4.4 ± 3.8	3.0 ± 2.9	2.3 ± 2.8^a^
	Worn Out	0.7 ± 0.7	0.6 ± 0.6	0.4 ± 0.6
	Listless	0.6 ± 0.6	0.3 ± 0.5	0.3 ± 0.6
	Fatigued	0.8 ± 0.7	0.7 ± 0.8	0.3 ± 0.6^a^
	Exhausted	0.5 ± 0.6	0.4 ± 0.6	0.2 ± 0.4
	Sluggish	1.0 ± 0.8	0.6 ± 0.6	0.5 ± 0.5^a^
	Weary	0.6 ± 0.7	0.4 ± 0.6	0.3 ± 0.5^a^
	Bushed	0.5 ± 0.7	0.3 ± 0.5	0.2 ± 0.6^a^
Vigor	Overall Morning	8.5 ± 3.9	9.1 ± 4.7	9.0 ± 4.6
	Overall Afternoon	8.0 ± 4.0	10.1 ± 4.1^b^	10.8 ± 4.3^a^
	Lively	1.2 ± 0.8	1.2 ± 0.7	1.5 ± 0.7
	Active	0.7 ± 0.8	1.0 ± 0.7	1.4 ± 0.7^a^
	Energetic	0.9 ± 0.8	1.2 ± 0.7	1.2 ± 0.7
	Cheerful	1.5 ± 0.7	1.8 ± 0.6	1.8 ± 0.6^a^
	Alert	1.3 ± 0.8	1.5 ± 0.6	1.7 ± 0.5^a^
	Full of Pep	0.6 ± 0.6	0.9 ± 0.7^b^	1.0 ± 0.7^a^
	Carefree	1.3 ± 0.7	1.5 ± 0.7	1.3 ± 0.8
	Vigorous	0.4 ± 0.6	0.4 ± 0.7	1.0 ± 0.7^a^
FCQ	Breakfast	47 ± 10	44 ± 10^b^	44 ± 9^a^
	Lunch	45 ± 12	44 ± 11	42 ± 9^a^
	Snack	40 ± 10	39 ± 9	38 ± 8
Urinary hormone concentrations	Norepinephrine (μg/g)	34.1 ± 10.6	37.5 ± 10.6	39.3 ± 11.3
	Epinephrine (μg/g)	6.5 ± 2.8	6.7 ± 3.7	8.0 ± 4.8
	Cortisol (μg/L)	9.2 ± 4.1	8.3 ± 7.3	8.4 ± 4.0
	Dopamine (μg/g)	180.8 ± 53.3	172.7 ± 47.6	186.4 ± 62.1

Mean +/- SD, ^a^ *P* < 0.05 SIT versus MICRO, ^b^ *P* < 0.05 SIT versus ONE

*SIT* uninterrupted sitting condition, *ONE*, uninterrupted sitting plus one continuous 30-min bout of moderate intensity treadmill walking, *MICRO*, uninterrupted sitting plus six 5-min bouts of moderate intensity treadmill walking, performed every hour for 6 h

### Cognitive performance

No significant effects of condition (SIT, ONE, MICRO) were observed for flanker task reaction time (ms) for incongruent trials (SIT: 460.14 ± 64.17; ONE: 453.89 ± 52.74; MICRO: 458.27 ± 54.39), congruent trials (SIT: 432.66 ± 62.08; ONE: 427.76 ± 55.74; MICRO: 428.38 ± 56.86), or for interference scores (SIT: 27.48 ± 17.94; ONE: 26.12 ± 18.10; MICRO: 29.89 ± 16.19). Furthermore, there were no significant effects of study condition on flanker task accuracy (% correct) for incongruent trials (SIT: 0.98 ± 0.03; ONE: 0.98 ± 0.03; MICRO: 0.98 ± 0.02), congruent trials (0.99 ± 0.01 for all conditions), or for interference scores (SIT: −0.01 ± 0.02; ONE: −0.01 ± 0.03; MICRO: −0.01 ± 0.03). Similarly, there were no significant effects of condition on CTMT composite index scores (SIT: 54.00 ± 10.30; ONE: 54.30 ± 10.39; MICRO: 54.90 ± 10.21).

### Appetite ratings

**Fig. 4:**
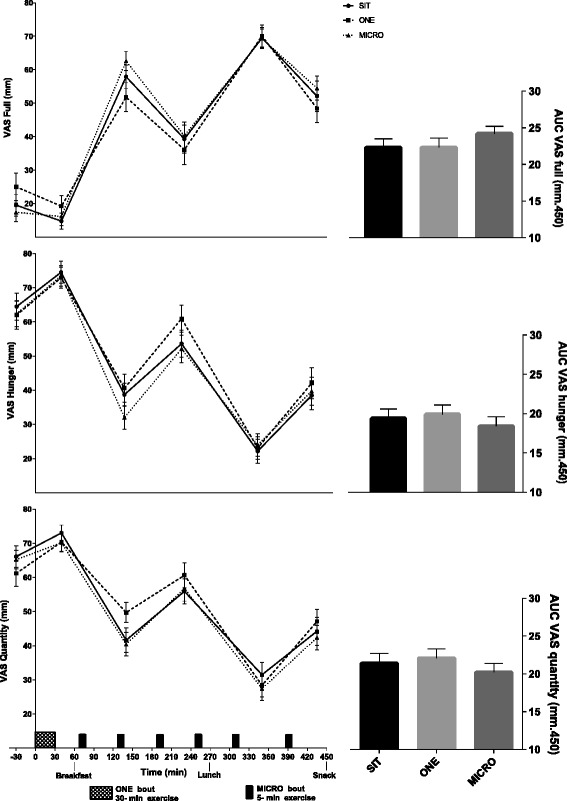
Self-perceived fullness (*Top panel*), hunger (*Middle panel*) and desire to eat (*Bottom panel*) over the day (*Left*) and area under the curve (AUC; *Right*) in uninterrupted sitting (SIT), uninterrupted sitting plus one 30-min continuous bout of moderate intensity treadmill walking (ONE) and uninterrupted sitting plus six 5-min bouts of moderate intensity treadmill walking, performed every hour for 6 h (MICRO), in healthy adults (*n* = 30). Changes over the day and between conditions, as well as differences in AUC, were tested by using a linear mixed model

The pattern of appetite ratings across the day is illustrated in Fig. 4. In all three conditions, participants reported feeling more full less hungry and had a decreased desire to consume food (main effect of time: *p* < 0.0001 for all) by the end of the day as compared to the start of the day. There were no significant differences between conditions on the evolution of appetite measures across the day. No statistical differences were noted between the SIT, MICRO and ONE conditions on perceived fullness, hunger or desire to eat food AUCs. While FCQ scores were not significantly different when measured before breakfast and before the snack between the three conditions, participants reported significantly reduced food cravings before lunch in the MICRO compared to SIT condition (Table 3, *p* = 0.01).

We observed a number of significant associations between energy levels and mood and feelings of hunger, fullness and the desire to consume food among the different conditions and time points. Overall, there were correlations between perceived energy levels and perceived hunger and desire to eat. Even more consistent were the relationships between POMS-F scores obtained at the end of the day and FCQ scores. Significant correlations were found between perceived fatigue and food cravings in the SIT (POMS-F vs. FCQ_lunch_, *r* = 0.38, *p* = 0.04), ONE (POMS-F vs. FCQ_lunch,_ *r* = 0.46, *p* = 0.01) and MICRO (POMS-F vs. FCQ_snack_, *r* = 0.48, *p* = 0.01) conditions. In the MICRO condition, energy level AUC was negatively associated with FCQ_breakfast_ (*r* = −0.40, *p* = 0.03), FCQ_lunch_ (*r* = −0.40, *p* = 0.03) and FCQ_snack_ (*r* = −0.40, *p* = 0.03).

### Urinary measures

There were no significant differences in urinary epinephrine, norepinephrine, cortisol and dopamine between conditions (Table 3).

While no significant associations were observed in either SIT or ONE conditions, we observed significant negative correlations between urinary cortisol and both perceived mood AUC (*r* = −0.39, *p* = 0.04) and POMS-V scores (*r* = −0.37, *p* = 0.05) in the MICRO condition. We further observed that changes induced by MICRO condition compared to SIT in epinephrine were positively correlated to changes in mood AUC between MICRO and SIT (*r* = 0.41, *p* = 0.03).

## Discussion

This is the first study to examine, under controlled laboratory conditions, the impact of physical activity performed as one single continuous bout or as multiple short bouts spread out across the day on energy levels, mood, fatigue and cognitive performance, compared to uninterrupted sitting in healthy adults. Both physical activity interventions replaced time spent seated by time spent walking at moderate intensity. Both interventions improved self-perceived energy levels over the day and vigor at the end of the day, compared to uninterrupted sitting. The multiple short bouts of activity furthermore improved mood throughout the day and reduced feelings of fatigue in the late afternoon. Overall, microbouts of activity led to sustained effects along the day, while the effects of the single bout of activity performed early in the morning did not last throughout the day. Finally, neither of the exercise regimens altered cognitive performance. This study provides the first evidence that microbursts of activity during the day improve energy level, mood and fatigue level, while maintaining usual levels of cognitive function.

This study provides the first evidence that microbursts of activity during the day improve energy level, mood and fatigue level, while maintaining usual levels of cognitive function [24–26]. Most previous studies thus far have tested the effect of use of standing desk workstations and of frequent transitions from sitting to standing position in either laboratory or office environments. In laboratory conditions, Thorp et al. [24] showed that transitioning from a sitting to standing position every 30 min for 4 days promoted concentration, alertness, motivation and activity, but demonstrated no clear improvement in productivity. The use of height-adjustable workstations that allow workers to transition seamlessly between seated to upright postures have also been shown to reduce feelings of fatigue [26]. In a randomized, cross-over trial, it was shown that the use of sit-stand desks reduced time spent sitting at work by 21 % while increasing energy and overall sense of well-being, and decreasing fatigue, with no impact on productivity [25]. A recent 8-week brisk walking intervention in sedentary employees of a high-tech company improved subjective fatigue, motivation and concentration [27], further showing that such interventions are feasible in ‘real world’ settings and provide similar beneficial effects on overall well-being as those observed in laboratory conditions.

The current study did not observe any changes in cognitive function in either of the exercise conditions. Previous studies have found that single 20- or 30-min bouts of exercise acutely improve cognitive performance immediately post-exercise [28–31]. However, we did not find that 30 min of exercise performed as either a single bout in the morning, or as multiple bouts throughout the day, was sufficient to improve cognitive performance measured at the end of the day. The fact that neither exercise condition was associated with detrimental effects on performance supports the feasibility of including such interventions in workplace environments. Furthermore, it is possible that the regular use of exercise microbursts throughout the day over a longer period of time may beneficially impact cognitive function. Future longer term studies could address this important question. It is also possible that practice effects masked intervention effects on cognitive performance. Given the potential for effects of learning, we chose to administer the cognitive tests on each of the three study days, but not at baseline. It does appear, however, that there may have been practice effects on the CTMT, with significant improvements observed with each administration of the task, ignoring intervention assignment (*p* < 0.001). Practice effects were not observed with the Flanker task. Intervention order was counterbalanced across participants in an attempt to overcome potential practice effects, but it is possible that improvement across repeated task administration may have masked intervention effects for the CTMT. A possible future approach could be to administer this test multiple times at baseline to minimize future practice effects, as has been suggested previously [32].

Compared to sitting, the greater average in daily heart rate measured in both physically active conditions suggests that stimulation of blood flow may help with alertness and maintenance of energy levels, mood and vigor [33, 34]. Although no statistical differences were noted in stress hormones between the three conditions, the relationships observed between cortisol and both perceived mood and vigor scores, as well as between epinephrine and mood also suggest that the benefits on overall well-being provided by the performance of microbursts of activity may be associated with prevention of physiological stress. Perceived fatigue was further associated with food cravings, which was reduced when time spent sitting was broken up. This result is consistent with the reduced appetite and dietary intake reported by office workers using the sit-stand workstations [25]. Replacing sitting time with moderate-intensity activity may suppress hunger or buffer the desire to eat. In fact, physical activity has been hypothesized to decrease appetite through endocrine mechanisms, thus reducing caloric intake [35]. Even though the impact of microbouts of activity on appetite and feeding behavior was small, this may have promising clinical implications for weight management in the general population, given that energy imbalance of only 50–100 kcal [36] can result in weight gain over time.

Office workers are one occupational group particularly vulnerable to prolonged and uninterrupted sedentary behavior [37]. The notion of an intervention that can improve employee well-being and performance has attracted interest from occupational health and human resources professionals. Although active workstations have demonstrated some promising and positive effects, they are very expensive and therefore cannot be implemented on a large scale. Even if active workstations reduce sedentary behavior that has been recognized as an independent health risk factor, they cannot allow the user to reach moderate-intensity activity as recommended by public health authorities. Brisk walking, like that performed in the current study, requires no special skills or expensive equipment, and can be performed anywhere at any time [38]. Interestingly, we observed that some beneficial effects of physical activity were more sustained across the day when the activity was broken up into multiple short bouts of activity performed across the day than when performed as a single continuous bout before the workday. In addition, observational studies have shown that time spent sitting, independent of levels of moderate-to-vigorous physical activity, are positively correlated with the risk of diabetes, cardiovascular disease, some cancers and premature mortality [39–41]. Because those who spend more time sitting at work may also spend more time sitting during leisure time [9], strategies to prevent sedentary behaviors at work like the one tested in this study may have important health implications in the general population.

A major strength of the current study was that it was conducted as a randomized, controlled trial under supervised laboratory conditions, which meant we were able to ensure full compliance from study participants. We further adopted a thorough examination of the effects of physical activity on well-being and cognitive performance by combining behavioral questionnaires, objective measures of cognitive function, measures of hormonal surrogates of physiological stress, and potential confounding factors, such as appetite. A limitation of the study, as for most lifestyle interventions, is that the intervention could not be blinded and primary outcomes were self-assessed by participants. It is possible that the wide broadcast of the health implications associated with sedentary behavior in the media may have biased participants’ responses towards the physically active conditions. However, we adjusted for period and sequence in the statistical analyses and implemented a 1-week ‘washout’ between study visits to minimize carry-over effects. The restriction of the study to healthy, non-obese individuals, while necessary to minimize potential confounding influences, also limits the generalizability of our findings to the broader working population. Given the high prevalence of overweight and obesity in modern societies, understanding whether the observed benefits of microbursts of activity performed every hour on perceived energy levels, fatigue and mood can also be identified in at-risk populations should be the focus of future research. While hourly short bouts of activity may be more feasible for workers to perform than one longer bout, future studies conducted in real life work settings as opposed to laboratory environments will be needed to establish this.

## Conclusions and future directions

Office workers have been identified as one of the most vulnerable occupational groups for accumulating prolonged and uninterrupted sitting time. In this laboratory-based trial, we have demonstrated that introducing exercise microbursts across the day can reduce fatigue and improve energy level and mood, while maintaining usual cognitive performance. Contrary to effects following a single continuous bout of activity, the effects of microbursts of activity were sustained throughout the day. Based on these findings, occupational health initiatives may want to introduce physically active breaks during the workday routine, as they are likely to increase workers’ well-being and energy, without detrimentally impacting worker performance. Future studies are needed to confirm the efficacy of this intervention with large-scale, randomized, controlled trials assessing activity interventions in office environments and their effects on long-term productivity, cognitive performance, well-being and health outcomes.

## Abbreviations

AHWC: Anschutz Health and Wellness Center
AUC: Area under the curve
BDI-II: Beck depression inventory-II
BMI: Body mass index
CTMT: Comprehensive trail making test
FCQ: Food craving questionnaires
IPAQ: International physical activity questionnaire
MICRO: Sitting + microbursts of activity
ONE: Sitting + one bout of activity
POMS: Profile of mood states
RMR: Resting metabolic rate
SD: Standard deviation
SEM: Standard error of the mean
SIT: Uninterrupted sitting
VAS: Visual analog scale
